# Predicting healthcare professionals’ acceptance towards electronic personal health record systems in a resource-limited setting: using modified technology acceptance model

**DOI:** 10.1136/bmjhci-2022-100707

**Published:** 2023-03-06

**Authors:** Agmasie Damtew Walle, Tigist Andargie Ferede, Nebebe Demis Baykemagn, Aynadis Worku Shimie, Shimels Derso Kebede, Masresha Derese Tegegne, Sisay Maru Wubante, Chalachew Msganaw Yehula, Addisalem Workie Demsash, Mequannent Sharew Melaku, Muluken Belachew Mengistie

**Affiliations:** 1 Department of Health Informatics, Mettu University, Mettu, Ethiopia; 2 Department of Epidemiology and Biostatistics, University of Gondar, Gondar, Ethiopia; 3 Department of Health Informatics, University of Gondar, Gondar, Ethiopia; 4 Department of Health Informatics, Debre Markos University College of Health Science, Debre Markos, Ethiopia; 5 Department of Health Informatics, Wollo University, Dessie, Ethiopia

**Keywords:** electronic health records, public health informatics, record systems

## Abstract

**Objectives:**

Personal health record systems allow users to manage their health information in a confidential manner. However, there is little evidence about healthcare providers’ intentions to use such technologies in resource-limited settings. Therefore, this study aimed to assess predicting healthcare providers’ acceptance of electronic personal health record systems.

**Methods:**

An institutional-based cross-sectional study was conducted from 19 July to 23 August 2022 at teaching hospitals in the Amhara regional state of Ethiopia. A total of 638 health professionals participated in the study. Simple random sampling techniques were used to select the study participants. Structural equation modelling analysis was employed using AMOS V.26 software.

**Result:**

Perceived ease of use had a significant effect on the intention to use electronic personal health records (β=0. 377, p<0.01), perceived usefulness (β=0.104, p<0.05) and attitude (β=0.204, p<0.01); perceived ease of use and information technology experience had a significant effect on perceived usefulness (β=0.077, p<0.05); and digital literacy (β=0.087, p<0.05) and attitude had also a strong effect on intention to use electronic personal health records (β=0.361, p<0.01). The relationship between perceived ease of use and the intention to use was mediated by attitude (β=0.076, p<0.01).

**Conclusion:**

Perceived ease of use, attitude and digital literacy had a significant effect on the intention to use electronic personal health records. The perceived ease of use had a greater influence on the intention to use electronic personal health record systems. Thus, capacity building and technical support could enhance health providers’ acceptance of using electronic personal health records in Ethiopia.

**WHAT IS ALREADY KNOWN ON THIS TOPIC**

- Adopting a sustainable electronic personal health records (ePHRs) in Ethiopia is challenged by a lack of top-level commitment and a physician-led aversion to using the system.
- A personal health record system is a crucial intervention for various health management purposes.
- For effective implementation of a personal health record system, considering acceptance to use the system is crucial.

**WHAT THIS STUDY ADDS**

- This study introduces a modified technology acceptance model.
- This study assessed users’ acceptance in Ethiopia, which aided in the development of a locally relevant automated record system for Ethiopia’s healthcare system improvement.
- The results of this study were used as input to design and test the effectiveness of a locally developed record system in Ethiopia.

**HOW THIS STUDY MIGHT AFFECT RESEARCH, PRACTICE OR POLICY**

- The findings may alleviate any concerns about the acceptance of personal health records, and because there is limited evidence on the acceptance of personal health records, it serves as a baseline for researchers in a resource-limited setting. Practically, this study offers insights for policymakers, developers, managers and decision-makers in the healthcare industry to improve the use and acceptability of the ePHRs.

## Introduction

Over the past decades, a variety of eHealth technologies have been accessible as nations have implemented eHealth efforts to support the objectives for health education and person-centred care.^1^ Adoption of personal health records (PHRs) has been linked to numerous advantages, including improved patient–provider relationships, patient engagement improvements, better medication adherence, good health outcomes (such as blood pressure and glycaemic management) and higher organisational efficiencies.^2^ Even though PHRs are intended to be consumer-oriented tools, simply understanding the consumer’s perspective is not enough. Although these issues have gotten less attention, consumer PHR use has significant consequences for healthcare providers and delivery systems as well.^3^ The value that consumers obtain from using a PHR will probably be directly influenced by the acceptance and behaviours of healthcare professionals and team members within the context of the clinical setting, despite the fact that PHRs have received a lot of attention as tools to help consumers.^4^Although electronic PHRs (ePHRs) have a great deal of potential to enhance healthcare, there are obstacles to their widespread implementation.^5^ Despite general agreement on the advantages of ePHRs, healthcare professionals have not been made aware of or receptive to this technology.^6^ According to preliminary research findings in the literature, patient adoption of a PHR may be influenced by provider endorsement, and continuing physician involvement in patient PHR use may be necessary to achieve and maintain predicted good health outcomes.^7^ Providing proper control for patient information disclosure and finding out how to process potentially enormous amounts of self-reported data within the constrained time allotted for the clinical visit are healthcare providers’ tasks.^8^

In Ethiopia, eHealth has been developing slowly. Technology problems, a lack of government support and budget over-runs are few of the reasons for this slow progress.^9^ Given this, ePHRs are not widely used and accessible electronic records are likewise reluctant to catch on. The most significant factors influencing health providers’ support for a national patient portal were expected positive influences on their work, the usability of the portal and benefits for the patients, according to a study conducted in Finland with a wide range of health providers (including nurses, pharmacists, health officers, doctors, physical therapists and psychologists).^10^

In Ethiopia, there is little evidence about acceptance of healthcare professionals of using PHR system to change the current healthcare system through eHealth technologies. The study may have effects on practice, policy and upcoming researches. Accordingly, this study investigates, introduces and empirically tests a modified theoretical model based on technology acceptance model (TAM) to identify the main factors influencing healthcare professionals’ acceptance of using ePHRs.

### Theoretical background and hypothesis

Several models have been used to predict factors associated with the acceptance of health information system technologies.^11 12^ The TAM is primarily applied at the individual level (but can also be applied in organisational settings), whereas Unified Theory of Acceptance and Use Technology 2 is primarily applied at the organisational level one of the most used models, and focuses on factors influencing end users’ behavioural intentions to use new technologies.^13 14^

**Figure 1:**
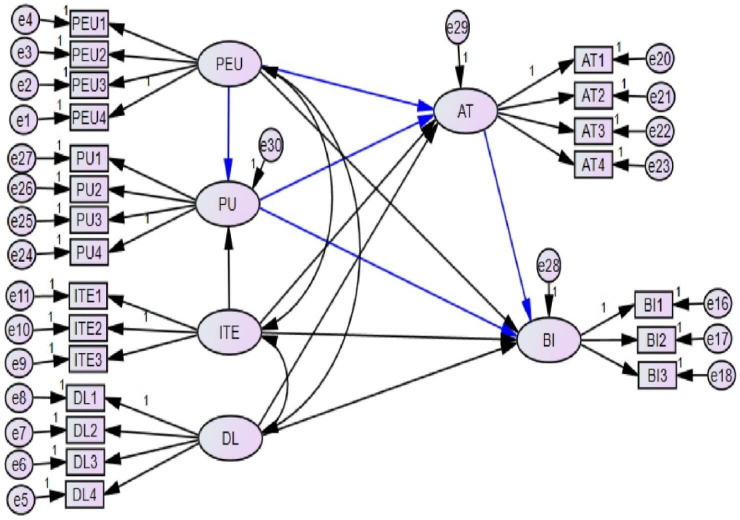
The original model (blue lines) and the modification proposed in this study (black lines). AT, attitude; BI, behavioural intention; DL, digital literacy; ITE, information technology experience; PEU, perceived ease of use; PU, perceived usefulness.

Perceived usefulness (PU) and perceived ease of use (PEU) are considered to be the main factors that either directly or indirectly determine behavioural intentions to use or embrace new technology in TAM.^13 15^ In this study, we included information technology and digital literacy components to measure the behavioural intention of health professionals to use ePHRs in low-resource settings. Since the actual use of ePHRs in the setting was unclear, the construct ‘actual use’ was not used (figure 1).

The following parts provide an explanation of the research question hypotheses that were produced for this study’s examination based on our model, which we adopted.

### Perceived usefulness

PU describes how much users believe the new technology will help them in their jobs, and studies showed that PU influences acceptance of using ePHRs.^15 16^ Based on those findings, the following hypotheses were tested.

H1: PU has a positive influence on the user’s attitudes towards ePHRs.

H2: PU has a positive influence on intention to use ePHRs.

H3: PU mediates the relationship between PEU and attitude towards ePHRs.

H4: PU mediates the relationship between information technology experience (ITE) and attitude towards ePHRs.

### Perceived ease of use

PEU is the degree to which a person believes that using technology will be simple and easy, and studies showed that PEU influences acceptance of using ePHRs.^13 15 16^ The following hypotheses were tested.

H5: PEU has a positive influence on the perceived usefulness of ePHRs.

H6: PEU has a positive influence on the user’s attitudes towards ePHRs.

H7: PEU has a positive influence on intention to use ePHRs.

### Attitude

Attitude exhibits how individuals’ thoughts toward a new technology affect their feelings and behaviour, and studies showed that attitude influences acceptance of using ePHRs.^13 16^ This study tests the following hypotheses:

H8: attitude towards eHealth positively influences intention to use ePHRs.

H9: attitude mediates the relationship between PU and intention to use ePHRs.

H10: attitude mediates the relationship between PEU and intention to use ePHRs.

H11: attitude mediates the relationship between ITE and intention to use ePHRs.

H12: attitude mediates the relationship between digital literacy and intention to use ePHRs.

### Information technology experience

ITE focuses on the information technology expertise of healthcare professionals, their exposure to technology and their comprehension of its fundamental advantages, and studies showed that ITE influences acceptance of using ePHRs.^13 17^ This study tests the following hypotheses:

H13: healthcare providers’ ITE has a positive influence on users’ PU of ePHRs.

H14: healthcare providers’ ITE has a positive influence on attitude towards ePHRs.

H15: healthcare providers ITE has a positive influence on intention to use ePHRs.

#### Digital literacy

Digital literacy describes a person’s capacity to seek, evaluate, and communicate information using writing and other media across a range of digital platforms, and influences acceptance of using ePHRs.^18^ The following hypotheses were examined in this study:

H16: healthcare providers’ digital literacy has a positive influence on attitudes toward ePHRs.

H17: healthcare providers’ digital literacy has a positive influence on intention to use ePHRs.

## Methods

### Study design and setting

An institution-based cross-sectional study was employed to determine health professionals’ acceptance of using ePHR and its predictors in the University of Gondar and Tibebe-Ghion Specialized Teaching Hospital in Amhara regional state, Ethiopia from July 19 to August 23 2022.

### Study participants and sample size determination

All healthcare professionals who worked in Amhara regional state teaching hospitals were the source population, whereas healthcare professionals who worked in Amhara regional state teaching hospitals during the study period were the study population. A 1:10 ratio of respondents to free parameters to be estimated was suggested for the estimation of sample size based on the number of free parameters in the hypothetical model.^19^ As a result, taking participants to a free parameter’s ratio of 10, a non-response rate of 10% and the 58 parameters were estimated based on the hypothesised model. Finally, a sample size of 638 was calculated.

### Sampling procedure

Participants in the study were selected from the University of Gondar and Tibebe-Ghion Specialized Teaching Hospital, located in the northwestern part of Amhara regional state of Ethiopia using a simple random sampling method.

### Data collection tools, procedures and data quality control

In this study, we applied a standard questionnaire, which is adapted from the original instrument developed by Davis’s study and previous studies of the modified TAM.^13 15 16 18^ The questionnaire consists of sociodemographics, TAM constructs (PU, PEU, attitude and behavioural intention), and additional elements of ITE and digital literacy. The constructs were measured using a 5-point Likert scale, in which 1 denotes ‘strongly disagree’ and 5 denotes ‘strongly agree’.^20^

The survey was a self-administered questionnaire. Two days of training were given for data collectors and supervisors. Pretesting of the questionnaire was conducted among 10% of the total study participants outside the study. After obtaining feedback from the respondents, language experts modified the wording of the questions and verified the internal consistency of the items using the Cronbach’s alpha coefficient, composite reliability and standard loading. The three tests’ results showed that all of the items’ scores were above the standard, so the original data collection was continued.

### Data processing and analysis

To analyse descriptive data, respondents’ data were entered into Epi-info V.7 and exported to SPSS V.25. Model constructs were assessed by the structural equation modelling analysis using Analysis of Moment Structure (AMOS) V.26 software.

Confirmatory factor analysis with standardised values was applied to the test the measurement model. To examine the goodness of fit, we used the Χ^2^ ratio (≤5), Tucker-Lewis index (TLI >0.9), comparative fit index (CFI >0.9), the goodness-of-fit index (GFI >0.9), adjusted GFI (AGFI >0.8), root mean square error approximation (RMSEA <0.08) and standardised root mean squared residual (SRMR <0.08).^16^

Construct reliability was evaluated using Cronbach’s alpha test and composite reliability, with each construct in the study reaching the necessary threshold of 0.70.^21^ Convergent validity was determined using the average variance extracted (AVE) method, with values greater than 0.5 and item loading greater than 0.6, and divergent validity was examined using the Fornell-Larcker criterion; the root of the AVE for a particular construct is greater than its correlation with all other constructs.^16^ The relationship between exogenous and endogenous variables was assessed using squared multiple correlations (R^2^), the critical ratio and the path coefficient to test a structural model. The statistical significance of the predictors was determined using 95% CIs and a p value of <0.05.

As indicated in table 1, each of the model’s six potential mediation paths was examined for its impact and level of significance. When a construct’s direct, indirect and total effects are all significant, it is referred to as partial mediation, but when direct and indirect effects are significant but the total effect is insignificant, that is referred to as full mediation. Generally, we considered a significant indirect effect with a p value of <0.05 to confirm mediation.

**Table 1:** Mediating effects of attitude (AT) and perceived usefulness (PU), and predicting health professionals’ acceptance of using electronic personal health record systems in a resource-limited setting, 2022

Path	Hypothesis	Effect	β	P value	Result	Decision
PEU→PU→AT	H3	Total	0.212	0.000***	Direct relationship	Not supported
		Indirect	0.008	0.072		
		Direct	0.204	0.000***		
ITE→PU→AT	H4	Total	0.035	0.216	No relationship	Not supported
		Indirect	0.006	0.068		
		Direct	0.029	0.301		
PU→AT→BI	H9	Total	0.025	0.614	No relationship	Not supported
		Indirect	0.029	0.093		
		Direct	−0.003	0.912		
PEU→AT→BI	H10	Total	0.454	0.000**	Partial mediation	Support
		Indirect	0.076	0.000**		
		Direct	0.377	0.000**		
ITE→AT→BI	H11	Total	−0.014	0.652	No relationship	Not supported
		Indirect	0.012	0.256		
		Direct	−0.027	0.326		
DL→AT→BI	H12	Total	0.107	0.010*	Direct relationship	Not supported
		Indirect	0.019	0.185		
		Direct	0.087	0.029*		

*Significance at p<0.05, **significance at p<0.01,*** significance at p<0.001.

BI, behavioural intention; DL, digital literacy; Hn, hypothesis; ITE, information technology experience; PEU, perceived ease of use; β, estimate.

## Results

### Sociodemographic characteristics of healthcare professionals

A total of 638 study subjects were included in the study; 610 (response rate: 95.61%) of them gave their consent and responded to the questions. Of the total n=610 respondents, 344 (56.4%) of them were male, and almost half of the respondents (313; 51.3%) had less than or equal to 3 years of work experience. Two hundred eighty-seven (47%) of the respondents had a Bachelor of Science degree, around two-thirds of the respondents (67.5%) had used social media and around three-fourths (455; 74.6%) of the participants had taken basic computer training. In addition, the median age of the respondents was 31.5 (IQR: 27–38) years (table 2).

**Table 2:** Sociodemographic characteristics of healthcare professionals in a resource-limited setting, 2022

Sociodemographic characteristics	Category	Frequency (N)	Percentage
Gender	Male	266	43.6
	Female	344	56.4
Age (years)	≤29	239	39.2
	30–35	191	31.3
	>35	180	29.5
Educational status	Diploma	123	20.2
	Degree	287	47.0
	Master and above	200	32.8
Profession	Medical doctor	219	35.9
	Health officer	48	7.9
	Nurse	167	27.4
	Midwife	113	18.5
	Others*	63	10.3
Work experience	1–3	313	51.3
	4–7	107	17.5
	8–10	33	5.4
	>10	157	25.7
Computer training	Yes	455	74.6
	No	155	25.4
Social media use	Yes	412	67.5
	No	198	32.5

*Pharmacist, radiologist, anaesthetist and psychiatrist.

### Measurement model

#### Reliability and validity of the construct

As the results shown in table 3, using Fornell-Larcker criterion, the square root of the AVE of the construct or the bolded values (diagonal values) were higher than the other values in that column and row. As a result, the discriminant validity of the model’s constructs has been established.

**Table 3:** Discriminant validity between constructs for predicting healthcare professionals’ acceptance of using electronic personal health record systems in a resource-limited setting, 2022

Construct	PEU	DL	ITE	BI	AT	PU
PEU	0.823					
DL	−0.016	0.840				
ITE	−0.066	−0.014	0.883			
BI	0.429	0.111	−0.051	0.796		
AT	0.207	0.060	0.038	0.422	0.716	
PU	0.091	0.037	0.104	0.066	0.108	0.777

AT, attitude; BI, behavioural intention; DL, digital literacy; ITE, information technology experience; PEU, perceived ease of use; PU, perceived usefulness.

Table 4 demonstrates that all of the constructs have Cronbach’s alpha and composite reliability scores above 0.70. AVE scores were >0.5 and factor loading >0.6. Therefore, there was substantial convergent validity for all of the constructs.

**Table 4:** Convergent validity between constructs for predicting healthcare professionals’ acceptance of using electronic personal health record systems in a resource-limited setting, 2022

Construct	Indicators /items	Standard loading	Composite reliability	Cronbach’s alpha	AVE
Perceived usefulness (PU)	PU1	0.84	0.86	0.86	0.60
	PU2	0.84			
	PU3	0.67			
	PU4	0.74			
Perceived ease of use (PEU)	PEU1	0.89	0.89	0.90	0.68
	PEU2	0.90			
	PEU3	0.73			
	PEU4	0.76			
Information technology experience (ITE)	ITE1	0.79	0.91	0.91	0.78
	ITE2	0.96			
	ITE3	0.89			
Digital literacy (DL)	DL1	0.88	0.91	0.91	0.71
	DL2	0.84			
	DL3	0.83			
	DL4	0.81			
Behavioural intention (BI)	BI1	0.83	0.84	0.83	0.63
	BI2	0.71			
	BI3	0.83			
Attitude (AT)	AT1	0.71	0.81	0.80	0.51
	AT2	0.74			
	AT3	0.72			
	AT4	0.70			

AVE, average variance extracted.

### Goodness of fit

The findings demonstrate the following values: Χ^2^ difference=3.0, GFI=0.92, AGFI=0.89, CFI=0.95, TLI=0.94, RMSEA=0.06 and SRMR=0.04. Therefore, the goodness-of-fit model was achieved.

### Structural equation modelling

As indicated in table 5, the results showed that PEU had a significant effect on the intention to use ePHRs (β=0.377, 95% CI: 0.280, 0.475, p<0.01), PU (β=0.104, 95% CI: 0.001, 0.207, p<0.05) and attitude (β=0.204, 95% CI: 0.101, 0.306, p<0.01). Healthcare providers’ ITE had a significant effect on healthcare providers’ PU towards ePHRs (β=0.077, 95% CI: 0.019, 0.136, p<0.05). The user’s digital literacy had a significant effect on behavioural intention to use ePHRs (β=0.087, 95% CI: 0.009, 0.162, p<0.05), and attitude towards ePHRs is also found to have a strong effect on healthcare providers’ intention to use ePHRs (β=0.361, 95% CI: 0.263, 0.465, p<0.01).

**Table 5:** SEM analysis of predicting healthcare professionals’ acceptance of using electronic personal health record systems in a resource-limited setting, 2022

Path	Hypothesis	β	SE	CR	P value	95% CI		Decision
						Lower	Upper	
PU→AT	H1	0.079	0.046	1.730	0.099	−0.015	0.174	Not supported
PU→BI	H2	−0.013	0.042	−0.081	0.912	−0.097	0.088	Not supported
PEU→PU	H5	0.104	0.048	2.169	0.048*	0.001	0.207	Supported
PEU→AT	H6	0.204	0.048	4.238	0.000**	0.101	0.306	Supported
PEU→BI	H7	0.377	0.047	8.001	0.000**	0.280	0.475	Supported
AT→BI	H8	0.361	0.050	7.187	0.000**	0.263	0.465	Supported
ITE→PU	H13	0.110	0.031	2.480	0.010*	0.019	0.136	Supported
ITE→AT	H14	0.029	0.030	0.943	0.301	−0.028	0.086	Not supported
ITE→BI	H15	−0.027	0.028	−0.951	0.326	−0.076	0.026	Not supported
DL→AT	H16	0.053	0.040	1.317	0.194	−0.026	0.137	Not supported
DL→BI	H17	0.087	0.037	2.356	0.029*	0.009	0.162	Supported

*P<0.05, **p<0.001.

AT, attitude; BI, behavioural intention; CR, critical ratio; DL, digital literacy; Hn, hypothesis; ITE, information technology experience; PEU, perceived ease of use; PU, perceived usefulness; SEM, structural equation model; β, estimate.

**Figure 2:**
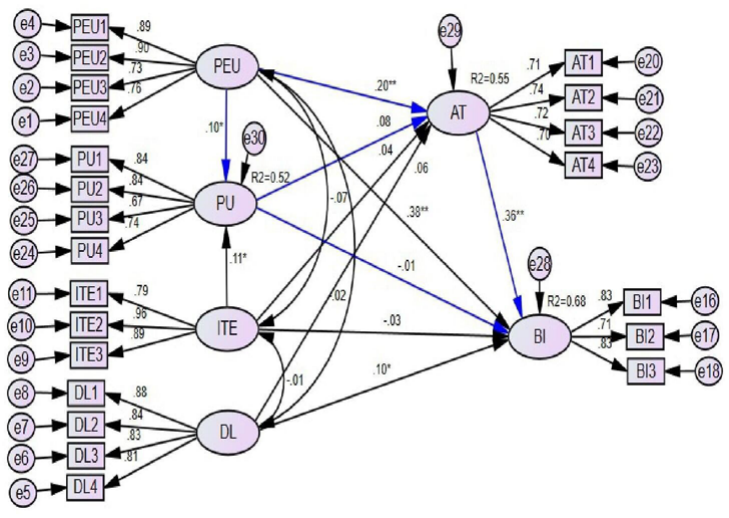
SEM analysis of predicting healthcare professionals’ acceptance of using electronic personal health record systems in a resource-limited setting, 2022. AT, attitude; BI, behavioural intention; DL, digital literacy; ITE, information technology experience; PEU, perceived ease of use; PU, perceived usefulness; SEM, structural equation modelling.

According to the findings, PU, PEU, ITE, and digital literacy accounted for 55% and 68% of the variance in attitude and intention to use ePHR systems, respectively (figure 2).

### Mediation effects

The results shown in table 1 indicated that the relationship between the PEU and the intention to use an ePHR was mediated by attitude. Both the relationship between attitude and intention to use ePHRs, as well as the regression coefficient between attitude and PEU, were statistically significant. The indirect effect’s standardised value was 0. 076. In the context of this, the indirect effect was statistically significant.

## Discussion

This study aimed to introduce modified TAM and determine factors influencing health providers’ acceptance in Ethiopia. As a result, H5, H6, H7, H8, H10, H13 and H17 were supported. The results showed that PEU had both direct and indirect implications on the desire to accept an ePHR, with favourable direct influences on PU and attitude. This indicated that when healthcare professionals assessed the system’s simplicity or ease of use, their perceptions of the system’s usefulness, attitude and intention to use it greatly improved. This result is consistent with results from other research conducted in various nations.^13 22 23^

This might be the case because the effort required to operate the system has a significant impact on a person’s attitude toward and acceptance of using ePHR systems. The effectiveness of the system will increase if it can more easily influence people’s inclinations to use ePHRs. To achieve long-term system acceptance, the system should be easy for healthcare practitioners.

Their tendency to use ePHR systems has been favourably impacted by the attitude of health professionals. This result is consistent with findings from related studies conducted in other settings.^1 24 25^ This may be due to the fact that new systems may irritate medical professionals who have a fixed, favourable opinion of ePHR systems. The availability of computers at work, continuing training and support, and knowledge sharing about eHealth technology are examples of actions that might be prioritised in order to change attitudes.

The intention to employ ePHRs was positively impacted directly by digital literacy. This proves that if healthcare workers were digitally literate, their intention to adopt technology would improve. The outcome is consistent with research done in various nations.^5 9 26^ The possible explanation could be due to the potential role of digital literacy in the adoption of digital health technologies being mandatory.

Users’ perceptions of the use of ePHRs were positively impacted by the ITE of healthcare practitioners. This demonstrates how healthcare professionals’ perceptions of the significance of new technology will change when they receive information technology skills and training from actual working environments. This result is consistent with research from other settings.^13 17^ The reason can be that experienced users are assured of their technical comprehension of what they need to pursue their hobbies, perform the tasks required to do so and readily realise how to improve their health. As a result, in order for end users to accept new technology and see its value, they must have extensive experience using it in environments with low resources.

### Limitations and future research

As a cross-sectional survey, the study might be biased toward social desirability. The quantitative study was not supported by the qualitative findings and the study only included teaching hospitals and required the inclusion of private, primary and general hospitals. As a result, future studies can employ a mixed-methods approach that incorporates qualitative and quantitative research techniques, and also add external variables that may influence the acceptance of using ePHRs, to gain a deeper understanding and more precise generalisation of the findings.

## Conclusion

This study examined factors affecting healthcare professionals’ acceptance of PHRs in Ethiopia. The proposed model has a strong ability to predict the hypothesised model because it could explain 68% of the variance in intention. PEU, attitude and digital literacy have a significant effect on the intention to use ePHRs, PU and attitude; healthcare providers’ ITE also has a significant effect on PU. PEU had a more significant impact on healthcare providers’ acceptance of using ePHR systems. Thus, capacity building and technical support could enhance health providers’ acceptance of using ePHRs in Ethiopia.

## Data Availability

All data relevant to the study are included in the article or uploaded as supplemental information.
